# Differential Impact of COVID-19 Risk Factors on Ethnicities in the United States

**DOI:** 10.3389/fpubh.2021.743003

**Published:** 2021-12-06

**Authors:** Prashant Athavale, Vijay Kumar, Jeremy Clark, Sumona Mondal, Shantanu Sur

**Affiliations:** ^1^Department of Mathematics, Clarkson University, Potsdam, NY, United States; ^2^Department of Biology, Clarkson University, Potsdam, NY, United States

**Keywords:** COVID-19, infection, mortality, ethnicity, Hispanic, risk factors, diabetes

## Abstract

The coronavirus disease (COVID-19) has revealed existing health inequalities in racial and ethnic minority groups in the US. This work investigates and quantifies the non-uniform effects of geographical location and other known risk factors on various ethnic groups during the COVID-19 pandemic at a national level. To quantify the geographical impact on various ethnic groups, we grouped all the states of the US. into four different regions (Northeast, Midwest, South, and West) and considered Non-Hispanic White (NHW), Non-Hispanic Black (NHB), Hispanic, Non-Hispanic Asian (NHA) as ethnic groups of our interest. Our analysis showed that infection and mortality among NHB and Hispanics are considerably higher than NHW. In particular, the COVID-19 infection rate in the Hispanic community was significantly higher than their population share, a phenomenon we observed across all regions in the US but is most prominent in the West. To gauge the differential impact of comorbidities on different ethnicities, we performed cross-sectional regression analyses of statewide data for COVID-19 infection and mortality for each ethnic group using advanced age, poverty, obesity, hypertension, cardiovascular disease, and diabetes as risk factors. After removing the risk factors causing multicollinearity, poverty emerged as one of the independent risk factors in explaining mortality rates in NHW, NHB, and Hispanic communities. Moreover, for NHW and NHB groups, we found that obesity encapsulated the effect of several other comorbidities such as advanced age, hypertension, and cardiovascular disease. At the same time, advanced age was the most robust predictor of mortality in the Hispanic group. Our study quantifies the unique impact of various risk factors on different ethnic groups, explaining the ethnicity-specific differences observed in the COVID-19 pandemic. The findings could provide insight into focused public health strategies and interventions.

## 1. Introduction

Numerous researchers have found various comorbidities and other risk factors affecting the spread and prognosis of coronavirus disease (COVID-19). Recent work by many researchers has also demonstrated that the COVID-19 pandemic has affected marginalized ethnicities more severely. We thus hypothesize that the risk factors for COVID-19 must have affected different ethnic groups in a distinctive manner. In this paper, we aim to quantify the differential effect of risk factors on different ethnicities.

### 1.1. COVID-19 and Ethnicity

The public health crisis created by the COVID-19 has uncovered the historical inequalities (1–4) between ethnic groups in certain countries, in particular in the UK and US, which are countries with ethnically diverse populations. These observations and consistent fatal outcomes in the minority ethnic groups (5, 6) have led to speculations about why patients from these groups are susceptible to infections, followed by severe complications. These trends could be due to different rates of COVID-19 infections, underlying health conditions, living conditions including housing density, having jobs as essential workers, access to health care, quality of care, and a mixture of multiple factors among these groups. The United States national data (7) from states and municipalities reports disproportionate COVID-19 infections, hospitalizations, and deaths among minority ethnic groups. Dobin and Dobin (8) showed that the infection rate is 4-fold for the Black and Hispanic population in selected counties in New York state. Moore et al. (9) observe a disproportionate number of COVID-19 cases among underrepresented racial/ethnic groups in the United States. Adhikari et al. (10) show that the racial and ethnic disparities in COVID-19 infections and deaths existed beyond those explained by income inequality.

### 1.2. Effects of Geographical Location on COVID-19

The impact of COVID-19 varies widely across countries and even within a country or a region. For example, Sun et al. (11) showed a negative correlation between the number provincial COVID-19 cases and latitude, as well as altitude. Breen and Ermisch (12) use spatial autoregressive regression to show that the relation of COVID-19 mortality to social composition of geographical areas in England is distinct than that of non-COVID mortality. A number of factors including societal awareness and culture, public health measures, healthcare infrastructure, and more recently vaccination coverage are known underlie the variation for COVID-19 infection rates and adverse health outcome (13).

Although multiple studies have confirmed that black and Hispanic populations in the US are more vulnerable to COVID-19, to our knowledge, no data is available if they are equally susceptible across geographical locations. Stephens-Davidowitz (14) uses the search data from Google to show that there exists a wide variation in racism in the US within the 50 states. Thus, we surmise that the impact of the COVID-19 on various ethnicities may not be uniform in all regions of the US. Hence, we are interested in understanding if a geographical location plays a part in the variation of COVID-19 impact on minorities.

### 1.3. Comorbidities for COVID-19

Emerging evidence highlights that comorbid conditions such as obesity, cardiovascular disease (CVD), and type 2 diabetes are directly linked to the severity of the COVID-19 disease (15–17). A meta-analysis including 76,993 patients with COVID-19 showed diabetes, CVD, smoking, malignancy, chronic kidney disease, hypertension, chronic obstructive pulmonary disease (COPD) are associated with poor prognosis (18). This conclusion was further supported by Richardson et al. (19), and Sun et al. (20). Using logistic regression (21) show that obesity was a risk factor for the severity of the COVID-19 disease. Furthermore, in a retrospective cohort study, Busetto et al. (22) conclude that despite their young age, overweight patients were more likely to need assisted ventilation and access to intensive care units than patients with normal weight. The connection between obesity and pulmonary function is well-established, e.g., Sharp et al. (23) observe that obese patients have significantly decreased total respiratory compliance. Moreover, Li et al. (24) find that reduction in functional residual capacity and diffusion impairment are the most common abnormalities in obese patients. Yan et al. (18) show that diabetic patients experienced more mortality than non-diabetic patients. Finally, just as in the case of the SARS epidemic (25), COVID-19 has disproportionately affected the older population (26). In fact, in the US, 92% of the COVID-19 recorded deaths till June, 2020 are in the age group 55 years and above (27). In summary, the main comorbidities for COVID-19 include obesity, diabetes, advanced age, hypertension, and cardiovascular disease.

However, these risk factors affect different ethnicities differently. For example, Paeratakul et al. (28) find that among obese individuals, the prevalence of hypertension was higher in NHB subjects than other groups. Sturm and Hattori (29) observe that the prevalence of obesity is about double among NHB than among Hispanics or NHW. Kuzawa and Sweet (30) note that NHB suffer from a disproportionate burden of CVD relative to NHW. Thus, we are motivated to understand whether or not these comorbidities affect different ethnicities differently.

### 1.4. Impact of Poverty on COVID-19 Prognosis

Patel et al. (31) note that economically disadvantaged people are vulnerable to COVID-19 due to a combination of factors. A time-series analysis conducted by Elgar et al. (32) reveals that income inequality is associated with a higher number of deaths due to COVID-19 in 84 countries. In particular, in the US, the states with higher income inequality experienced a higher rate of infection as well as the number of COVID-19 related deaths (33). This pattern could be because the comorbidities associated with COVID-19 are linked to poverty.

A longitudinal study involving 600,662 adults from Taiwan's National Healthcare Insurance database indicates that diabetes incidence is associated with poverty (34). This finding is particularly notable since the subjects from this study had access to universal healthcare. However, the subjects were from a ethnically homogeneous population. Thus, we are motivated to investigate the differential role of poverty among various races.

### 1.5. Objectives

For this study, we choose Non-Hispanic white (NHW), Non-Hispanic Black (NHB), Hispanic, and Non-Hispanic Asian (NHA) as four ethnic groups. The risk factors we choose to focus on in this work are advanced age, obesity, cardiovascular disease, diabetes, hypertension, and poverty. We aim to investigate the following in this study:

1. Does a geographical location have different impact on COVID-19 infection and mortality rates for different ethnicities?
2. Do various COVID-19 risk factors have a different effect on different ethnicities?

To this end, we collected COVID-19 related infections and mortality data from various publicly available sources, as described in section 2.1. To investigate the geographical variation in the impact of COVID-19, we seek to quantify the difference between the distributions of infection rates, mortality rates, and populations of various ethnic groups in four geographical regions of the US (35). We describe our approach for this analysis in section 2.4.1. Finally, in section 2.4.3 we construct robust linear models with infection and mortality rates of various ethnicities as response variables.

## 2. Materials and Methods

### 2.1. Selection of Variables and Data Sources

Given the discussion in the sections 1.3, 1.1, and 1.4, we focus on the following factors in this work: obesity, diabetes, poverty, advanced age, hypertension, and cardiovascular diseases. We included the following ethnic groups in our work: NHW, NHB, Hispanic, and NHA. We excluded the groups American Indian/Alaska Native, and Native Hawaiian/Other Pacific Islander from this work due to lack of reliable and consistent data sets in the US, cf. (36–38). We used the American Community Survey (39) census database to collect the following information for each state: NHW, NHB, Hispanic, and NHA population and their respective percentage contribution to the population of each state. To investigate the geographical effect on COVID-19 prognosis, we used the classification of the US into the following categories: Northeast, South, Midwest, and West (35). COVID-19 infection and death counts between January 21, 2020, to September 30, 2020 from KFF Covid-19 data (40) were obtained. The time window roughly corresponds to the first pandemic wave experienced in the US. For each ethnicity **E**, and for a specific state **S** the variable Relative Infection % is defined as follow:


$$\begin{array}{l} \text{Relative Infection \%}\\ :=\frac{\text{COVID-19 positive subjects of ethnicity }\text{E}\text{ in state}\text{S}}{\text{Total number of COVID-19 positive subjects in state }\text{S}}\times 100. \end{array}$$

We note that the choice of “relative” infection percentage as a response variable is deliberate. This choice allows us to directly compare this number with the population share of that ethnicity in the region. Similarly, for each ethnicity **E**, and for a specific state **S** we define the variable Relative Mortality % as follow:


$$\begin{array}{l} \text{Relative Mortality \%}\\ :=\frac{\text{COVID-19 related deaths for ethnicity }\text{E}\text{ in state }\text{S}}{\text{Total number of COVID-19 deaths in state }\text{S}}\times 100. \end{array}$$

In our work, Relative Infections % and Relative Mortality % were considered as the response variables. For brevity, we write infection rate instead of Relative Infections %, and so on. The use of relative percentages allows a direct comparison with the population percentages of that ethnicity. For example, in a state with a 5% NHB population, relative mortality of 15% in the NHB community indicates disproportionately large mortality compared to the NHB population. The use of “relative” percentage is independent of the population of the state itself. The use of this measure also allows us to compare the impact on a certain ethnic group in two states with similar proportion of the minority population. As a concrete example, when we consider the states of California and Texas, both have a similar percentage of the Hispanic population, 39.5 and 40%, respectively. However, the relative mortality percentages for the Hispanic group in California and Texas are 48.3 and 56.1%, respectively. We collected the data on the percentage of people with age 60 or more in each state and ethnicity is obtained from the CDC dataset (41). Race and state-wise data were obtained from adults who reported being told by a health professional that they have diabetes (excluding prediabetes and gestational diabetes) using the America's Health Rankings (42). We used the body mass index (BMI) as a measure of obesity following (43) and define obesity as a condition of having a BMI of 30.0 or higher. The dataset (44) were used to obtain the obesity data from each state and for the races NHW, NHB, Hispanic, NHA. We acquired the percentage of adults whom a health care professional informed that they had a coronary heart disease, or myocardial infarction, or a stroke from AHR CVD data (45). This was gathered for each state and ethnicity of interest. We obtained the race and state-wise data on adults who reported being informed by a health professional that they have high blood pressure from AHR HBP data (46). The US Census Bureau defines the “poverty threshold” for a family with two adults and one child as $20,578 in 2019. We extracted the data from KFF Poverty data (47) on poverty defined by the “poverty threshold.” We obtained this data for each state and ethnicity of interest.

For each state, and each of the four ethnicity of interest (NHW, NHB, Hispanic, and NHA) we defined the variables: Age60+, BMI30+ (a measure of obesity), CVD, Diabetes, HBP, Poverty. For a state **S**, and an ethnicity **E** we defined the relative percentage of people with age 60 or over Age60+ as follows:


$$\begin{array}{l} \text{Relative Age60+ \%}\\ :=\frac{\text{Number of people of ethnicity }\text{E}\text{ with age over 60 in state }\text{S}}{\text{Total number of people with age over 60 in state }\text{S}}\times 100. \end{array}$$

We use the variable name Age60+ instead of Relative Age60+ % for conciseness, and so on. We define the relative percentage variables Obesity, CVD, Diabetes, HBP, and Poverty in a similar manner.

### 2.2. A Note on Unavailability of Data From Some States

We encountered a few irregularities during our data collection process in the format the data was made available by various states (36–38). For example, New York state does not provide ethnicity-wise COVID-19 infection and mortality data. Similarly, infection data for NHW communities only are available for North Dakota. Data availability of specific variables for different ethnicities also varied across states. Data for all variables for the NHW group could be obtained from 48 states whereas for the NHB and Hispanic communities such data were available from only 38 and 33 states, respectively. In our analysis, we included the states for which data for all the variables are available. Hatcher et al. (37) find that only 23 state in the US have complete data for American Indian and Alaska Native Persons.

### 2.3. Description of Data

**Figure 1:**
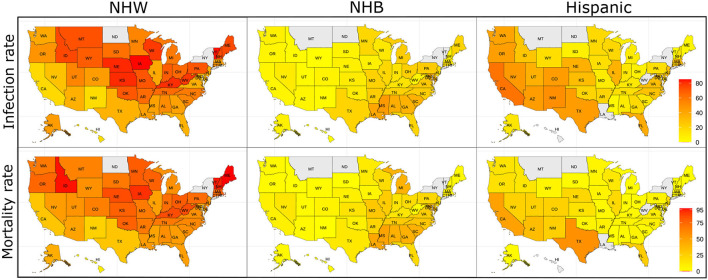
The relative infection % and relative mortality % amongst the NHW, NHB, and Hispanic groups across the US. The states with no color indicate that the ethnicity-wise data on relative infection % and relative mortality % were not available in those states.

**Figure 2:**
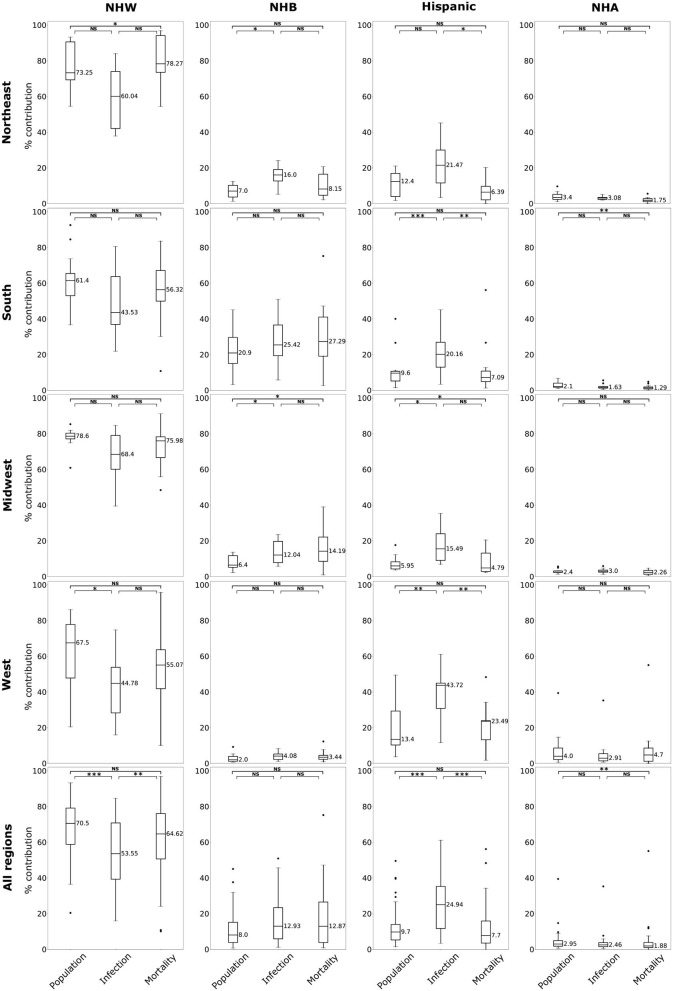
Box plots of population, relative infection %, and relative mortality % in each of four US regions, and combining all regions for NHW, NHB, Hispanic and NHA groups. Horizontal bars represent medians. “*” significance at *p* < 0.1, “**” significance at *p* < 0.05, “***” significance at *p* < 0.01, NS, not significant (Kruskal-Wallis tests followed by Dunn's tests).

The data for this study are state-level demographics based on four ethnic groups. We depict the relative infection % and relative mortality % for NHW, NHB, and Hispanic group in the map in Figure 1. As described in section 2.2 some states do not make the ethnicity-wise data public. The states with no color in the Figure 1 indicate that the ethnicity-wise infection and mortality data was not available in those states. We calculated state-wise descriptive statistics for the relative infection and mortality percentages and population comparing each ethnic group. We performed a descriptive analysis to explore the region-specific, state-wise characteristics of for the relative infection % and mortality % and population by calculating their medians, first and third quartiles, and presented in Figure 2.

### 2.4. Analytical Approach

#### 2.4.1. Quantifying the Regional Variability of COVID-19 on Various Races

The infection and mortality rates for various ethnic groups are disproportionate to their share of the population in the US (7, 8). We aim to understand this phenomenon and its severity across various regions in the US. To this effect, we employed the Kruskal-Wallis (KW) test (48), a non-parametric equivalent of the one-way analysis of variance. Since the test does not identify the groups that differ in their distributions, we followed it with Dunn's multiple comparisons test (49) for cases for which the KW test yielded statistically significant results. We used the combination of KW test, and Dunn's comparison test for the groups NHW, NHB, Hispanic, and NHA separately for all four regions of the US, as well as the whole country.

#### 2.4.2. Correlation Analyses

In order to quantify the association between the impact of COVID-19 on the ethnic groups and the risk factors across the country, we consider each state, for which the data are available, as a data point. We computed the pairwise Pearson's correlation coefficients between various risk factors for the racial groups NHW, NHB, and Hispanic, along with their 2-tailed statistical significance values. We summarized the comparisons between the variables in correlation matrices.

#### 2.4.3. Constructing Robust Linear Models With Infection and Mortality Rates as Response Variables

In order to elucidate the role of the explanatory variables on a specific aspect of the COVID-19 burden linear models are employed. For these linear models, we considered each state as a data point. From the Figure 2, we observe that the rate of infection and mortality in the NHA are consistently lower when compared to their population. Thus, we consider building linear models for NHW, NHB, and Hispanic groups only to elucidate the contributions of risk factors considered in this study. However, infection and mortality rates, the response variables for our model, showed skewness in their distributions. Since logarithmic (log) transformation of data is one of the most commonly used techniques to conform to normality (50), we implemented it on infection and mortality rates. The log transformation was effective in correcting the skewness and introduce normality (Supplementary Figures S1, S2). As log transformation of infection and mortality rates improved their normality behavior, we used log transformed form of these variables exclusively for model construction. Thus, when we refer to infection rate and mortality rate in the context of linear models they denote log transformed infection rate and mortality rate, respectively. For NHW, NHB, and Hispanic groups, we built our preliminary linear models with infection rate, and mortality rate as our response variables and the risk factors defined in section 1.3, i.e., advanced age Age60+, BMI30+ (a measure of obesity), CVD, Diabetes, HBP, Poverty as the explanatory variables.

However, conditions of advanced age, cardiovascular disease, diabetes, obesity, and hypertension are interrelated. This interrelation can also be observed from the correlation **Tables 3–5**. Multicollinearity among the explanatory variables can lead to unstable and unreliable estimates of regression coefficients (51). We used the variance inflation factor (VIF) to assess the multicollinearity between the explanatory variables (52). Following Kutner et al. (53, p. 409) an upper cut-off value of VIF for explanatory variables is set as 10 to minimize the contribution of multicollinearity in our model. Starting from the preliminary model for ethnicity of interest, we propose the procedure outlined below to construct our final model:

1. Compute the VIF for each explanatory variable in the model. If all the VIFs are less than 10, we declare this to be the final linear model.
2. If an explanatory variable has a VIF of more than 10, we remove the explanatory variable with the largest VIF. If there are more than one explanatory variables with VIF within 5% of the maximum VIF, we remove the variable that leads to a model with the highest adjusted *R*^2^.
3. We construct the linear model with the remaining explanatory variables. After the removal of a variable, it is possible to include more data points in our model. For example, after removing the variable Diabetes, we could include states for which data on diabetes was not available.
4. Go to Step 2.4.3.

After constructing the linear models, we checked the normality of the residuals of the regression models with Lilliefors normality test (54).

### 2.5. Geographically Weighted Regression

Linear regression yields stationary and global regression coefficients. However, it is conceivable that these coefficients might have local variability. To find the geographical variability in the coefficients, we employed the geographically weighted regression (GWR) (55). Rather than producing global regression results, GWR yields “local” regression coefficients in terms of geographically varying functions. For our analysis, we used the infection rate and mortality rates as response variables and the variables obtained from section 2.4.3 as the explanatory variables.

### 2.6. Coding Language and Libraries Used

For our coding, we used R language (version 4.0.0), along with the following libraries in our coding: readxl, dplyr, tidyr, FSA, ggplot2, car, qqplotr, nortest, pwr, spgwr, sp, sf, rgdal, rgeos, tmap, tmaptools.

## 3. Results

### 3.1. Regional Variation of COVID-19 Impact on Various Ethnicities

The boxplots in Figure 2 summarize the relative impact of COVID-19 on various ethnicities across the four regions of the US and all regions as an aggregate. In Figure 2 we present various descriptive statistics of the population, infection, and mortality rates for the NHW, NHB, Hispanic, and the NHA groups across various regions. As noted in the section 2.2, not all the states are included in the analyses. Thus, the statistics shown in this plot do not correspond closely to those of the whole country. We describe the Kruskal-Wallis test results in Table 1. We see in Table 1 that the KW test for NHW is statistically significant in the Northeast and the West with *p* < 0.1. For the NHB group, the KW test is significant in the Northeast and the Midwest with *p* < 0.1. The KW test was statistically significant for the Hispanic group in “all four regions,” with *p* < 0.1 in the Northeast; with *p* < 0.05 in Midwest and the West; and *p* < 0.01 in the South. The NHA data yielded significant results with the KW test only in the South with *p* < 0.05.

**Table 1:** Non-parametric Kruskal-Wallis test for each ethnic group's relative infection %, relative mortality %, and population percentages per region as groups and their significance levels.

	**NHW**		**NHB**		**Hispanic**		**NHA**	
	**Statistic**	***p*−value**	**Statistic**	***p*−value**	**Statistic**	***p*−value**	**Statistic**	***p*−value**
Northeast	5.38*	0.068	5.43*	0.066	5.21*	0.074	3.81	0.148
South	4.37	0.112	2.95	0.228	10.96***	0.004	7.77**	0.020
Midwest	3.88	0.137	5.83*	0.054	8.66**	0.013	2.40	0.302
West	4.88*	0.087	2.02	0.364	8.83**	0.012	0.60	0.741
All regions	13.03***	0.002	4.83*	0.089	26.18***	<0.01	5.38*	0.067

“*” *significance at p < 0.1*,

“**” *significance at p < 0.05*,

“***” *significance at p < 0.01*.

When we considered all four regions in the US together, the KW test was statistically significant for all ethnicities with *p* < 0.01 for NHW and Hispanic communities. The KW test was significant for the NHB and NHA when all regions were combined with *p* < 0.1.

We followed the significant KW tests with Dunn's multiple comparison test to identify factors differing in their distributions. We depict the results from the Dunn's test in Table 2. In particular, we obtained statistically significant results (with *p* < 0.05) in the South, Midwest, and West for the Hispanic population between the pairs ‘infection & mortality rates' and “infection rate and population share.”

**Table 2:** *Post-hoc* analysis using Dunn Test and their significance levels.

	**Infection%-Mortality%**		**Infection%-Population**		**Mortality%-Population**	
**Ethnic group-region**	**Statistic**	***p*−value**	**Statistic**	***p*−value**	**Statistic**	***p*−value**
NHW-Northeast	−2.30*	0.064	−1.41	0.314	0.88	0.377
NHW-South	–	–	–	–	–	–
NHW-Midwest	–	–	–	–	–	–
NHW-West	−1.09	0.275	−2.21*	0.081	−1.12	0.527
NHW-All	−2.29**	0.043	−3.55***	0.001	−1.26	0.207
NHB-Northeast	1.29	0.392	2.32*	0.060	1.03	0.301
NHB-South	–	–	–	–	–	–
NHB-Midwest	−0.19	0.842	1.09*	0.094	2.18*	0.087
NHB-West	–	–	–	–	–	–
NHB-All	0.37	0.711	2.06	0.117	1.69	0.181
Hispanic-Northeast	2.27*	0.068	1.31	0.375	−1.02	0.305
Hispanic-South	2.88**	0.011	2.87***	0.008	−0.10	0.917
Hispanic-Midwest	2.67**	0.022	2.42**	0.031	−0.31	0.753
Hispanic-West	2.33**	0.039	2.78**	0.015	0.36	0.718
Hispanic-All	4.60***	<0.01	4.28***	<0.01	−0.47	0.638
NHA-Northeast	–	–	–	–	–	–
NHA-South	1.22	0.221	−1.55	0.239	−2.78**	0.016
NHA-Midwest	–	–	–	–	–	–
NHA-West	–	–	–	–	–	–
NHA-All	1.39	0.325	−1.30	0.190	−2.7**	0.020

“*” *significance at p < 0.1*,

“**” *significance at p < 0.05*,

“***” *significance at p < 0.01*.

### 3.2. Results of the Correlation Analyses

The KW test provides evidence of geographical impact on various ethnicities. In this section we provide the results of correlation analysis between other risk factors. In Table 3 we see the Pearson correlations between the variables along with the 2-tailed significance values for the NHW group. The same statistics are provided in Tables 4, 5 for the NHB and Hispanic communities respectively. All the variables are strongly (*p* < 0.01) and positively correlated with every other variable for all ethnicities, with poverty being the sole exception. To be precise, for the NHW group, poverty is positively correlated with the infection rate (*r* = 0.35, *p* < 0.05), and diabetes (*r* = 0.29, *p* < 0.05). Poverty is either uncorrelated or weakly correlated with other variables in this study for all ethnicities.

**Table 3:** Pearson correlations for NHW ethnic group between variables used in the study and their significance levels.

		**Infections%**	**Mortality%**	**Poverty**	**Age60+**	**Diabetes**	**BMI30+**	**HBP**	**CVD**
Infection%	Pearson correlations	1							
	Sig. (2-tailed)								
Mortality%	Pearson correlations	0.82***	1						
	Sig. (2-tailed)	<0.01							
Poverty	Pearson correlations	0.35**	0.17	1					
	Sig. (2-tailed)	0.014	0.230						
Age60+	Pearson correlations	0.82***	0.91***	0.16	1				
	Sig. (2-tailed)	<0.01	<0.01	0.267					
Diabetes	Pearson correlations	0.78***	0.83***	0.29**	0.94***	1			
	Sig. (2-tailed)	<0.01	<0.01	0.042	<0.01				
BMI30+	Pearson correlations	0.88***	0.91***	0.21	0.96***	0.90***	1		
	Sig. (2-tailed)	<0.01	<0.01	0.141	<0.01	<0.01			
HBP	Pearson correlations	0.86***	0.91***	0.20	0.98***	0.93***	0.99***	1	
	Sig. (2-tailed)	<0.01	<0.01	0.178	<0.01	<0.01	<0.01		
CVD	Pearson correlations	0.84***	0.91***	0.21	0.98***	0.93***	0.97***	0.98***	1
	Sig. (2-tailed)	<0.01	<0.01	0.148	<0.01	<0.01	<0.01	<0.01	

“*” *significance at p < 0.1*,

“**” *significance at p < 0.05*,

“***” *significance at p < 0.01*.

**Table 4:** Pearson correlations for NHB ethnic group between variables used in the study and their significance levels.

		**Infection%**	**Mortality%**	**Poverty**	**Age60+**	**Diabetes**	**BMI30+**	**HBP**	**CVD**
Infection%	Pearson correlations	1							
	Sig. (2-tailed)								
Mortality%	Pearson correlations	0.93***	1						
	Sig. (2-tailed)	<0.01							
Poverty	Pearson correlations	−0.03	−0.07	1					
	Sig. (2-tailed)	0.833	0.677						
Age60+	Pearson correlations	0.90***	0.95***	−0.10	1				
	Sig. (2-tailed)	<0.01	<0.01	0.550					
Diabetes	Pearson correlations	0.92***	0.95***	−0.084	0.99***	1			
	Sig. (2-tailed)	<0.01	<0.01	0.610	<0.01				
BMI30+	Pearson correlations	0.94***	0.96***	−0.09	0.99***	0.99***	1		
	Sig. (2-tailed)	<0.01	<0.01	0.592	<0.01	<0.01			
HBP	Pearson correlations	0.94***	0.96***	−0.09	0.99***	0.99***	0.99***	1	
	Sig. (2-tailed)	<0.01	<0.01	0.592	<0.01	<0.01	<0.01		
CVD	Pearson correlations	0.86***	0.92***	0.07	0.99***	0.98***	0.96***	0.97***	1
	Sig. (2-tailed)	<0.01	<0.01	0.691	<0.01	<0.01	<0.01	<0.01	

“*” significance at p < 0.1, “**” significance at p < 0.05,

“***” *significance at p < 0.01*.

**Table 5:** Pearson correlations for Hispanic ethnic group between variables used in the study and their significance levels.

		**Infection%**	**Mortality%**	**Poverty**	**Age60+**	**Diabetes**	**BMI30+**	**HBP**	**CVD**
Infection%	Pearson correlations	1							
	Sig. (2-tailed)								
Mortality%	Pearson correlations	0.84***	1						
	Sig. (2-tailed)	<0.01							
Poverty	Pearson correlations	−0.16	−0.14	1					
	Sig. (2-tailed)	0.306	0.349						
Age60+	Pearson correlations	0.74***	0.82***	−0.07	1				
	Sig. (2-tailed)	<0.01	<0.01	0.632					
Diabetes	Pearson correlations	0.83***	0.85***	−0.04	0.96***	1			
	Sig. (2-tailed)	<0.01	<0.01	0.793	<0.01				
BMI30+	Pearson correlations	0.85***	0.87***	−0.16	0.97***	0.99***	1		
	Sig. (2-tailed)	<0.01	<0.01	0.319	<0.01	<0.01			
HBP	Pearson correlations	0.80***	0.81***	−0.11	0.98***	0.98***	0.98***	1	
	Sig. (2-tailed)	<0.01	<0.01	0.482	<0.01	<0.01	<0.01		
CVD	Pearson correlations	0.75***	0.82***	−0.01	0.98***	0.97***	0.98***	0.98***	1
	Sig. (2-tailed)	<0.01	<0.01	0.950	<0.01	<0.01	<0.01	<0.01	

“*” significance at p < 0.1, “**” significance at p < 0.05,

“***” *significance at p < 0.01*.

### 3.3. Results of the Linear Models With Infection and Mortality Rates as Response Variables for Each Ethnic Group

In Table 6 we depict the linear models with infection rate and mortality rate as response variables for NHW. The first column shows preliminary models along with the VIFs for each explanatory variable. The second column depicts the final model obtained via the maximum VIF elimination algorithm described in section 2.4.3. The preliminary model with infection rates in the NHW community as the response variable accounts for 83% [$R^{2}=0.83,R_{adj}^{2}=0.80,F_{\left(6,41\right)}=32.87,p<0.01$] of the variability in the infection rates for NHW population. The final model for the NHW infection rates consists of obesity, diabetes, and poverty as the only explanatory variables. This model accounts for 82% [$R^{2}=0.82,R_{adj}^{2}=0.80,F_{\left(3,44\right)}=65.06,p<0.01$] of the variability in the NHW infection rates. The final NHW infection model and preliminary model both use 48 states.

**Table 6:** Linear models with infection and mortality rates as response variables for NHW.

**Linear regression models withs infection rate in NHW population as a response variable**						
	**Preliminary model**			**Final model**		
	**β (95*%CI*)**	***Pr* (> *t*)**	**VIF**	**β (95*%CI*)**	***Pr* (> |*t*|)**	**VIF**
(Intercept)	2.21 (1.53, 2.89)	<0.01***		2.46 (2.16, 2.74)	<0.01***	
BMI30+	0.04 (0.01, 0.06)	<0.01***	59.51	0.02 (0.02, 0.03)	<0.01***	5.35
Diabetes	−0.002 (−0.01, 0.01)	0.956	9.67	−0.004 (−0.01, 0.03)	0.318	5.47
Poverty	0.02 (−0.00, 0.05)	0.067*	1.20	0.02 (−0.00, 0.05)	0.082*	1.09
Age60+	0.20 (−0.01, 0.05)	0.787	58.57			
HBP	−0.03 (−0.06, 0.01)	0.725	104.88			
CVD	−0.01 (−0.03, 0.01)	0.309	39.07			

	$R^{2}=0.83,R_{adj}^{2}=0.80,n=48$			$R^{2}=0.82,R_{adj}^{2}=0.80,n=48$		
	*F*_(6, 41)_ = 32.87, *p* <0.01***			*F*_(3, 44)_ = 65.06, *p* <0.01***		

**Linear regression models with mortality rate in NHW population as a response variable**						
	**Preliminary model**			**Final model**		
	**β (95*%CI*)**	***Pr* (> *t*)**	**VIF**	**β (95*%CI*)**	***Pr* (> |*t*|)**	**VIF**

(Intercept)	1.58 (1.03, 2.12)	<0.01***		2.59 (2.27, 2.91)	<0.01**	
BMI30+	0.003 (−1.65, 0.02)	0.785	59.51	0.86 (0.01, 0.02)	0.001***	5.35
Diabetes	−0.002 (−1.08, 0.01)	0.643	9.67	0.01 (0.00, 0.02)	0.039**	5.47
Poverty	0.01 (−1.38, 0.03)	0.512	1.20	−0.00 (−1.44, 0.98)	0.955	1.09
Age60+	0.04 (1.18, 0.06)	0.005***	58.57			
HBP	−0.03 (−5.40, 0.00)	0.070*	104.88			
CVD	0.02 (1.57, 0.04)	<0.050**	39.07			

	$R^{2}=0.88,R_{adj}^{2}=0.87,n=48$			$R^{2}=0.78,R_{adj}^{2}=0.77,n=48$		
	*F*_(6, 41)_ = 51.95, *p* <0.01***			*F*_(3, 44)_ = 53.48, *p* <0.01***		

*The first column shows preliminary model along with the VIFs for each explanatory variable. The second column indicates the model obtained using maximum VIF elimination algorithm*.

“*” *significance at p < 0.1*,

“**” *significance at p < 0.05, and*

“***” *significance at p < 0.01*.

The preliminary model with NHW mortality rates as the response variable accounts for 88% [$R^{2}=0.88,R_{adj}^{2}=0.87,F_{\left(6,411\right)}=51.95,p<0.01$] of the variability in the NHW mortality rates. The final model for the NHW mortality also consists of obesity, diabetes, and poverty as the only explanatory variables. This NHW mortality final model accounts for 7% [$R^{2}=0.78,R_{adj}^{2}=0.77,F_{\left(3,44\right)}=53.48,p<0.01$] of the variability in the NHW mortality.

In Table 7 we depict the linear models with infection rate and mortality rate as response variables for NHB. The preliminary model with infection rates in the NHB group as the response variable accounts for 81% [$R^{2}=0.81,R_{adj}^{2}=0.77,F_{\left(6,31\right)}=17.51,p<0.01$] of the variability in the infection rates for NHB population. The final model for the NHW infection rates consists of obesity and poverty as the only explanatory variables. This model accounts for 66% [$R^{2}=0.66,R_{adj}^{2}=0.64,F_{\left(2,37\right)}=37.89,p<0.01$] of the variability in the NHB infection rates. Note that the final NHB infection model uses 40 states instead of 38 states in the preliminary model. This discrepancy is because of the unavailability of data for the NHB community for all the explanatory variables, as discussed in section 2.4.3. The preliminary model with NHB mortality rates as the response variable accounts for 77% [$R^{2}=0.77,R_{adj}^{2}=0.73,F_{\left(6,31\right)}=17.51,p<0.01$] of the variability in the NHB mortality rates. The final model for the NHB mortality also consists of only obesity and poverty as the explanatory variables. This NHB mortality final model accounts for 67% [$R^{2}=0.67,R_{adj}^{2}=0.65,F_{\left(2,37\right)}=199.9,p<0.01$] of the variability in the NHB mortality rates.

**Table 7:** Linear models with infection and mortality rates as response variables for NHB.

**Linear regression models with infection rate in NHB population as a response variable**						
	**Preliminary model**			**Final model**		
	**β(95*%CI*)**	***Pr*(> *t*)**	**VIF**	**β(95*%CI*)**	***Pr*(> |*t*|)**	**VIF**
(Intercept)	1.10 (0.42, 1.79)	0.002**		2.12 (1.42, 2.84)	<0.01***	
BMI30+	0.04 (−0.04, 0.13)	0.309	97.03	0.05 (0.04, 0.06)	<0.01***	1.00
Poverty	0.02 (−0.01, 0.05)	0.169	1.10	−0.01 (−0.04, 0.02)	0.39	1.00
Diabetes	0.02 (−0.06, 0.10)	0.695	101.10			
Age60+	−0.20 (−0.34, −0.05)	0.009***	164.01			
HBP	0.16 (0.06, 0.26)	0.003***	127.51			
CVD	−0.02 (−0.09, 0.05)	0.514	58.27			

	$R^{2}=0.81,R_{adj}^{2}=0.77,n=38$			$R^{2}=0.66,R_{adj}^{2}=0.64,n=40$		
	*F*_(6, 31)_ = 17.51, *p* <0.01***			*F*_(2, 37)_ = 37.89, *p* <0.01***		

**Linear regression models with mortality rate in NHB population as a response variable**						
	**Preliminary model**			**Final model**		
	**β (95*%CI*)**	***Pr* (> *t*)**	**VIF**	**β (95*%CI*)**	***Pr* (> |*t*|)**	**VIF**

(Intercept)	1.26 (0.36, 2.17)	0.008***		2.16 (1.32, 3.01)	<0.01***	
BMI30+	0.10 (−0.01, 0.22)	0.073*	97.03	0.06 (0.04, 0.07)	<0.01***	1.00
Poverty	0.00 (−0.04, 0.04)	0.921	1.10	−0.02 (−0.05, 0.12)	0.191	1.00
Diabetes	−0.04 (−0.15, 0.06)	439	101.10			
Age60+	−0.25 (−0.45, −0.06)	0.011**	164.01			
HBP	0.17 (−0.03, 0.30)	0.017**	127.51			
CVD	0.03 (−0.06, 0.12)	0.521	58.27			
	$R^{2}=0.77,R_{adj}^{2}=0.73,n=38$			$R^{2}=0.67,R_{adj}^{2}=0.65,n=40$		
	*F*_(6, 31)_ = 17.51, *p* <0.01***			*F*_(2, 37)_ = 199.9, *p* <0.01***		

*The first column shows preliminary model along with the VIFs for each explanatory variable. The second column indicates the model obtained using maximum VIF elimination algorithm*.

“*” *significance at p < 0.1*,

“**” *significance at p < 0.05, and*

“***” *significance at p < 0.01*.

The Table 8 depicts the linear models with infection rate and mortality rate as response variables for the Hispanic group. The preliminary model for infection rates in the Hispanic community accounts for 67% [$R^{2}=0.67,R_{adj}^{2}=0.60,F_{\left(6,26\right)}=8.97,p<0.01$] of the variability in the infection rates for the Hispanic population. The final model for the Hispanic infection rates consists of diabetes and poverty as the explanatory variables. This model accounts for 51% [$R^{2}=0.51,R_{adj}^{2}=0.48,F_{\left(6,25\right)}=9.98,p<0.01$] of the variability in the Hispanic infection rates. The preliminary model with mortality rates among the Hispanic community as the response variable accounts for 71% [$R^{2}=0.71,R_{adj}^{2}=0.63,F_{\left(6,25\right)}=9.98,p<0.01$] of the variability in the Hispanic mortality rates. The final model for Hispanic mortality consists of advanced age and poverty as the explanatory variables. Note that advanced age is the most significant explanatory variable in the final mortality model in the Hispanic group, whereas having diabetes was the most significant variable predicting infection in the Hispanic community. This final model accounts for 55% [$R^{2}=0.55,R_{adj}^{2}=0.53,F_{\left(2,39\right)}=23.93,p<0.01$] of the variability in the Hispanic mortality rates. We note that the final model for the Hispanic mortality includes 42 states, whereas the preliminary model has only 32 states due to lack of data availability.

**Table 8:** Linear models with infection and mortality rates as response variables for Hispanic.

**Linear regression models with infection rate in Hispanic population as a response variable**						
	**Preliminary model**			**Final model**		
	**β(95*%CI*)**	***Pr*(> *t*)**	**VIF**	**β(95*%CI*)**	***Pr*(> |*t*|)**	**VIF**
(Intercept)	2.32 (1.56, 3.083)	<0.01***		2.34 (1.63, 3.04)	<0.01***	
Diabetes	−0.03 (−0.11, 0.05)	0.439	44.72	0.04 (0.03, 0.052)	<0.01***	1.00
Poverty	0.00 (−0.04, 0.04)	0.931	1.04	0.01 (−0.02, 0.05)	0.489	1.00
BMI30+	0.15 (0.05, 0.24)	0.003***	74.89			
Age60+	−0.83 (−0.20, 0.04)	0.162	37.24			
HBP	0.02 (−0.10, 0.13)	0.783	61.21			
CVD	−0.08 (−0.18, 0.02)	0.113	40.27			

	$R^{2}=0.67,R_{adj}^{2}=0.60,n=33$			$R^{2}=0.51,R_{adj}^{2}=0.48,n=41$		
	*F*_(6, 26)_ = 8.97, *p* <0.01***			*F*_(2, 38)_ = 19.52, *p* <0.01***		

**Linear regression models with mortality rate in Hispanic population as a response variable**						
	**Preliminary model**			**Final model**		
	**β (95*%CI*)**	***Pr* (> *t*)**	**VIF**	**β (95*%CI*)**	***Pr* (> |*t*|)**	**VIF**

(Intercept)	2.07 (−0.96, 3.18)	0.001***		2.51 (1.60, 3.42)	<0.01***	
Age60+	−0.05 (−0.20, 0.10)	0.504	37.24	0.09 (0.06, 0.12)	<0.01***	1.00
Poverty	−0.04 (−0.10, 0.01)	0.138	1.04	−0.047 (−0.09, −0.00)	0.043**	1.00
Diabetes	−0.04 (−0.14, 0.06)	0.398	44.72			
BMI30+	151 (0.03, 0.27)	0.002**	74.89			
HBP	−0.02 (−0.16, 0.13)	0.828	61.21			
CVD	−0.04 (−0.17, 0.08)	0.487	40.27			
	$R^{2}=0.71,R_{adj}^{2}=0.63,n=32$			$R^{2}=0.55,R_{adj}^{2}=0.53,n=42$		
	*F*_(6, 25_) = 9.98, *p* <0.01***			*F*_(2, 39)_ = 23.93, *p* <0.01***		

*The first column shows preliminary model along with the VIFs for each explanatory variable. The second column indicates the model obtained using maximum VIF elimination algorithm*.

“*” significance at p < 0.1,

“**” *significance at p < 0.05, and*

“***” *significance at p < 0.01*.

**Figure 3:**
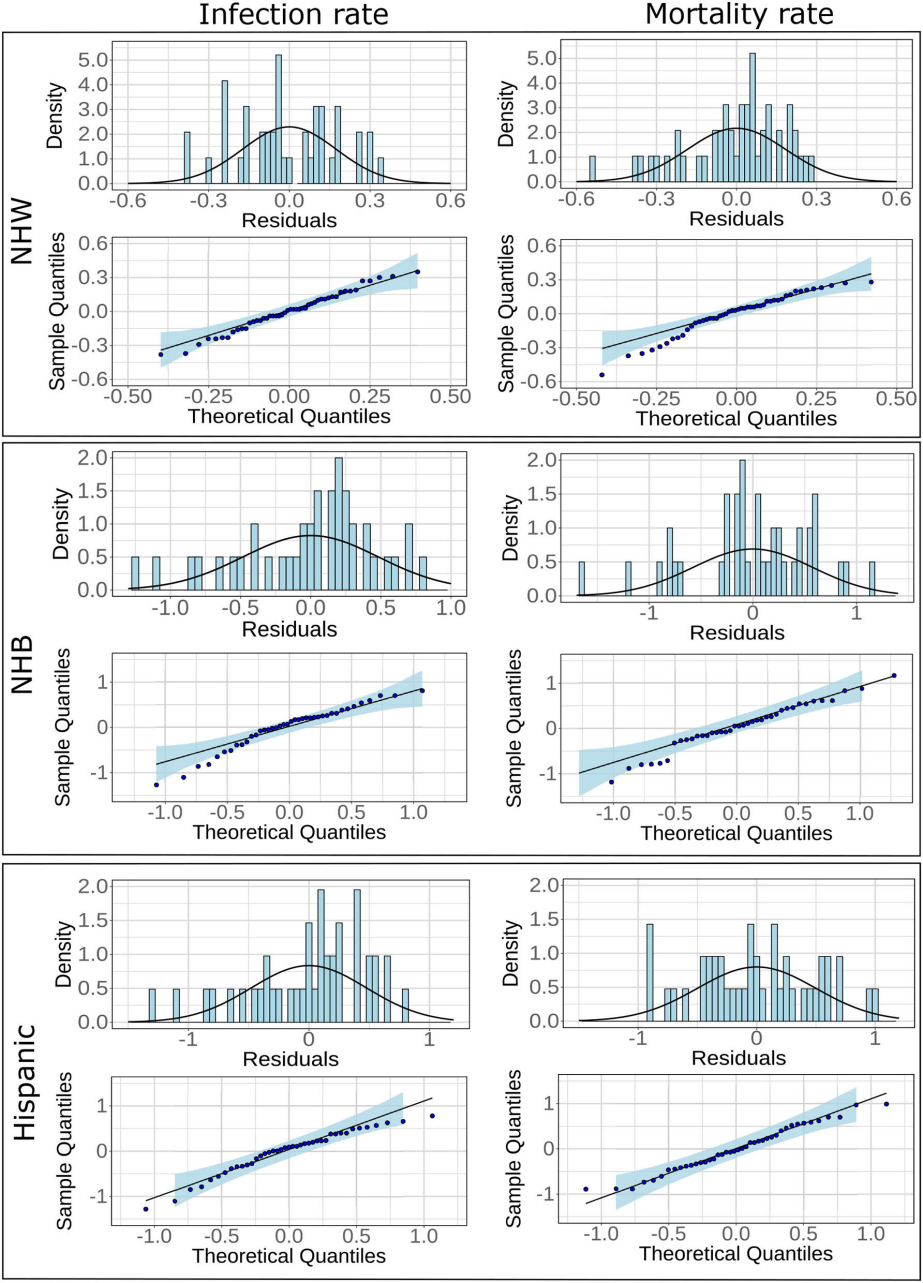
Histograms and fitted normal curves along with QQ plots of the residuals for linear regression for NHW, NHB, and Hispanics groups. The linear models are based on infections and mortality rates as response variables.

Adjusted *R*^2^ value for the regression model for NHW mortality was much higher (0.77) in comparison to NHB (0.65) and Hispanic (0.53). However, all six models showed statistical significance and satisfied normality tests for the residual values. Indeed, the Lilliefors normality test applied to the residuals obtained from each of these models revealed that the residuals were normally distributed with *p*>0.001. The histograms, and the QQ plots for the residuals are provided in Figure 3.

### 3.4. Results From the Geographically Weighted Regression

**Figure 4:**
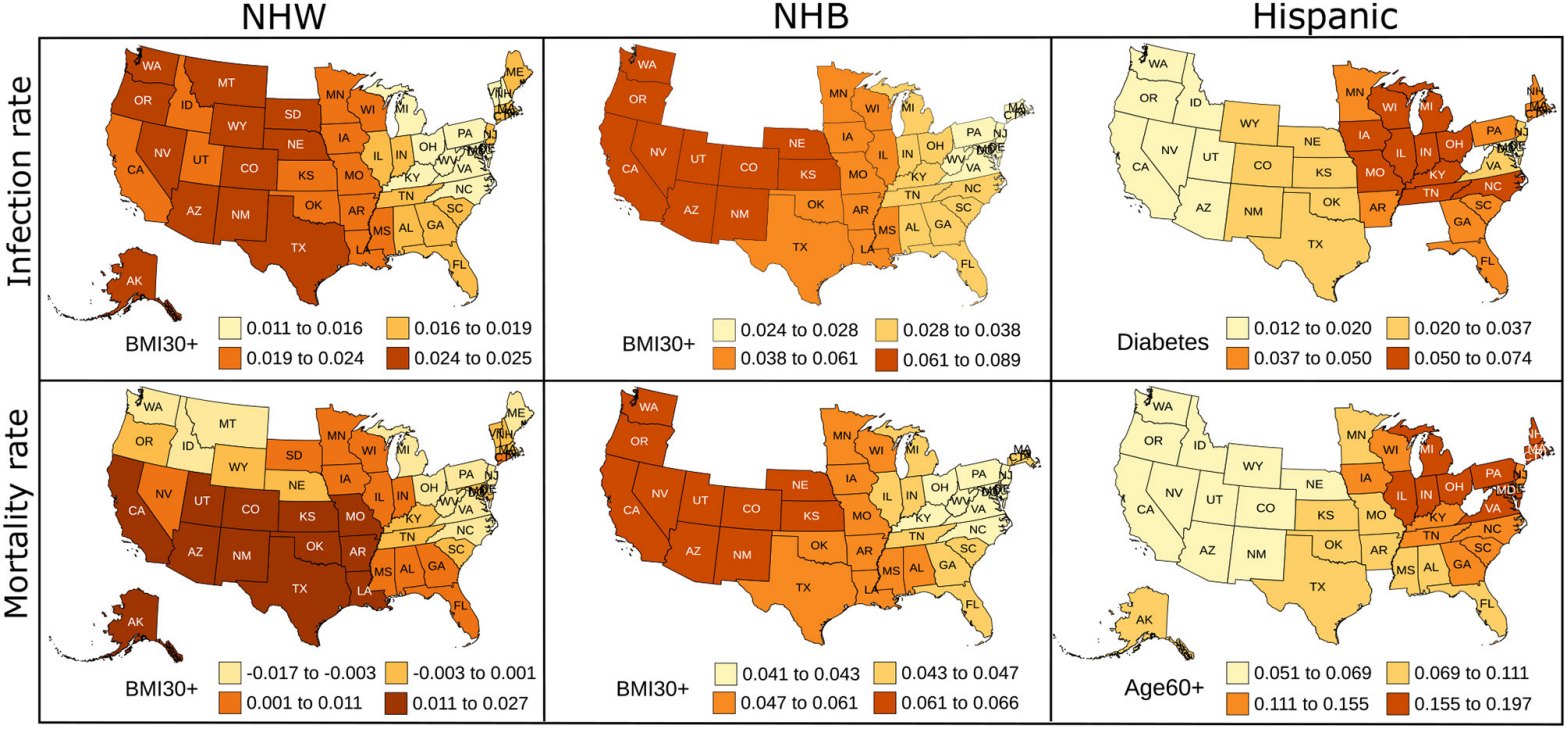
Map showing the coefficient values for the most significant variables for the GWR models constructed on predictors used in linear regression for NHW, NHB, and Hispanics data. The states with no color indicate that the ethnicity-wise data on the corresponding risk factors was not available in those states.

The geographically weighted regression yields coefficients for each risk factor for every state. We show the state-wise coefficients for the most significant explanatory variable for each ethnicity in Figure 4. Empty spaces for states in Figure 4 indicate that ethnicity-wise data was not available for those states for the corresponding risk factors. The *p*-values for the most significant explanatory variable and state-wise *R*^2^ values obtained from the GWR model are included in Supplementary Figures S4, S5.

## 4. Discussion

Our analysis of the nationwide data revealed that geographical location, and other COVID-19 risk factors affect different ethnicities in a dissimilar way. We observed that the disparate burden of the pandemic was most prominent on the NHB and Hispanic communities. This observation is supported by Anyane-Yeboa et al. (56) and Escoba et al. (57) other studies. In particular, the rate of infection was exceptionally high for the Hispanic community compared to their population share. Discordant impact on NHB and Hispanic populations has been reported by Centers for Disease Control (58) and studied using data from metropolitan cities and combining selected states, but the nationwide study is limited. In our work, this effect was observed in the four US regions separately and also when all the states' data was aggregated. When considered the four regions individually, we found that the excessive infection rate in the Hispanic community was most prominent in the South region. However, compared to the Hispanic group's infection rates, their mortality rates were statistically lower in all regions of the US. This apparent discrepancy could be because the Hispanic community is the youngest of the four ethnic groups considered in our study (59). The infection rate of NHB population was higher compared to their population share in the Midwest, and the Northeast than other regions.

The correlation analysis confirmed that the COVID-19 related risk factors such as advanced age, cardiovascular disease, diabetes, hypertension, and obesity are highly interrelated. This finding is consistent with numerous studies. For example, Mokdad et al. (60) show that obesity (BMI≥30) was significantly associated with diabetes, hypertension, high cholesterol, asthma, and arthritis. Wilson and Kannel (61) conclude that obesity and diabetes are associated with atherogenic risk factors. Abdullah et al. (62) also conclude that obesity is associated with type 2 diabetes. We also found that “within” an ethnic group, poverty was uncorrelated or weakly correlated with infections and mortality for all three ethnic groups, implying that poverty is an “independent” risk factor for COVID-19. This finding is supported by Elgar et al. (32) and Oronce et al. (33) which we discussed in section 1.4.

After eliminating variables with high multicollinearity, we formulated robust and parsimonious linear models for NHW, NHB, and Hispanic populations. The linear models described in section 3.3 reveal that “obesity” encapsulates many other co-dependent risk factors for the infection and mortality in NHW and NHB groups. This finding is expected in light of numerous studies (61, 63). Obesity and diabetes are well-established risk factors for COVID-19. In these two conditions, adipose tissue is compromised, which can directly or indirectly get involved in interaction with SARS-CoV-2, the pathogen responsible for COVID-19 disease (64). Thus, it is not surprising that obesity highly influences the regression models for NHW and NHB with death rate as a response variable. However, the degree of influence of obesity on infection rates and mortality is noteworthy, with obesity emerging as the most significant factor contributing to the infection rates and mortality for the NHW and NHB groups.

The Hispanic community markedly differs from NHW, and NHB with respect to the results of the linear models. Diabetes was the most significant factor for infection rate in Hispanics, while advanced age emerged as most significant for mortality. The effect of advanced age on Hispanic mortality could be also due to the relatively younger, and thus working-age, population of Hispanics (59) in the US.

The regression models indicate a strong association of poverty with a high infection rate, followed by death for all ethnic groups studied. This finding is in agreement with several studies focusing on the association of low socioeconomic status, which increases the exposure to COVID-19 (31, 65). People with low socioeconomic status avail healthcare services at an advanced stage of illness, thus experience a worse prognosis. The disease burden associated with obesity is linked to socioeconomic status and race (28). Ethnic minorities and populations with low socioeconomic status have been disproportionately affected in previous pandemics (5, 6, 8). Evidence from the COVID-19 pandemic is not an exception to the above fact. To this end, public health strategies to control the current and future pandemics need to take these ethnicity-specific effects into account to mitigate the spread and severity of the disease.

The linear regression furnishes global and static coefficients for the explanatory variables. However, the geographically weighted regression gives coefficients that are geographically varying. We see in Figure 4 the variability in the coefficients of the GWR. We note that the neighboring states seem to have similar coefficients, indicating similarity in the risk factors in nearby states. Obesity is the most prominent risk factor amongst the NHW and NHB populations, and diabetes and advanced age seem to be more influential in the Hispanic community. The GWR results for the Hispanic group show more variability than the NHW group, which could be due to the higher percentage of people of Hispanic origin in southern and western states. The local *R*^2^ map in Supplementary Figure S5 also indicates that the GWR model fits the NHW and NHB groups better than the Hispanic group. We plan to explore the geographical variation of the risk factors in more detail in future work.

### 4.1. Limitations

As discussed in section 2.2 and noted by other researchers (36, 38) there is a lack of consistency and availability of COVID-19 related data. Our study does not include data from the state of New York, since the state does not make the ethnicity wise data available. Moreover, the data we use is state wise statistics of the various risk factors. However, we note that the observations made using such data is consistent with those made by other researchers.

### 4.2. Practical Implications of the Study

Although racial and ethnic disparities in COVID-19 infections and mortality are becoming increasingly clear from several studies based on available data, drivers of these disparate outcomes remain less understood at a national level. Our models, based on the nationwide data, indicate that “obesity” effectively encapsulates the effect of other co-dependent factors for NHW, and NHB populations (section 3.3). The link between COVID-19 infection severity and obesity is noted by Watanabe et al. (66) even in the early stages of the pandemic. Similarly, during the H1N1 pandemic of 2009 (67, 68) observed that obesity was associated with higher mortality.

Another implication from our work is that the Hispanic community is more susceptible to the COVID-19 infection. This observation is valid throughout the US. This situation could be remedied via public policy changes and awareness of the issue. The disproportionate impact of COVID-19 on the minority population is largely attributed to existing socioeconomic inequities. The low-income minority population are often compelled to work in an environment with higher risk of disease exposure, live in a crowded accommodation, and lack adequate access to healthcare. The government support to low-income families in the form of the CARES Act, Consolidated Appropriations Act, 2021, Department of Treasury US (69) and the American Rescue Plan Act of 2021 (70) are critical but might not be sufficient to fully mitigate observed disparity in infection and mortality rates. Our analysis indicates that certain subpopulations of the minority population are at higher risk of COVID-19 infection and mortality. Identifying these vulnerable subpopulations, such as Hispanics with diabetes or age over 60 years, and prioritizing additional attention to these populations could enable a more efficient allocation and utilization of resources. Increased effort toward educating and raising awareness on COVID-19 and associated risk factors could also be an effective method to develop community resilience. One potential avenue to improve awareness on COVID-19 will be through recruiting volunteers to educate the vulnerable population. For example, “Philly counts” (71), a program supported by the Philadelphia Department of Public Health, initially created for Census 2020, currently helps direct community engagement efforts for the COVID-19 vaccine. Extending similar initiatives to populations with major risk factors such as obesity could result in a major beneficial impact on overall COVID-19 burden.

## 5. Conclusion

Several researchers have concluded that several health conditions, poverty, and geographical location affect the COVID-19 prognosis. Studies have shown that the COVID-19 pandemic has impacted some minorities in the US more severely than other groups. Our work focused on quantifying this distinct effect of various COVID-19 risk factors on different ethnicities in the US during the first pandemic wave.

To this effect, we included Non-Hispanic White, Non-Hispanic Black, Hispanic, Non-Hispanic Asians. Our work has revealed differences in the way the COVID-19 pandemic affected various ethnic groups. We observed that the infection rates in the Hispanic population were disproportionately larger than the share of their population across all regions of the US. This effect was most prominent in the South region. The NHA populations consistently had lower infection rates and mortality rates compared to their population. Furthermore, we studied the following risk factors in this work: advanced age, obesity, cardiovascular diseases, diabetes, hypertension, and poverty for NHW, NHB, and Hispanic populations. We aimed to quantify the different effects of these risk factors on various ethnicities. To this end, we constructed linear models with infection and mortality rates as the response variables. We eliminated variables causing multicollinearity from our models, leading to robust linear models. Our models indicate that “obesity” parsimoniously describes the impact of other co-dependent comorbidities for NHW and NHB populations (section 3.3). However, for the infection rates in the Hispanic group, the factor leading to the robust linear model was the prevalence of diabetes. On the other hand, advanced age was more significant for COVID-19 related mortality for the Hispanic community. We also established “poverty” as an independent risk factor for infection and mortality amongst the three ethnicities: NHW, NHB, and Hispanics. The findings in this study quantified ethnicity-specific effects of COVID-19 risk factors, which we hope could be mollified with public policy interventions and community engagement.

## Data Availability Statement

Publicly available datasets were analyzed in this study. This data can be found here: https://github.com/prashantva/Covid-19-Ethnicity.

## Author Contributions

PA: writing—review, conceptualization, editing, investigation, and analysis. JC: data collection. VK: data curation, formal analysis, visualization, and coding. SM: writing—original draft, methodology, formal analysis, and project administration. SS: supervision, conceptualization, validation, and editing. All authors contributed to the article and approved the submitted version.

## Conflict of Interest

The authors declare that the research was conducted in the absence of any commercial or financial relationships that could be construed as a potential conflict of interest.

## Publisher's Note

All claims expressed in this article are solely those of the authors and do not necessarily represent those of their affiliated organizations, or those of the publisher, the editors and the reviewers. Any product that may be evaluated in this article, or claim that may be made by its manufacturer, is not guaranteed or endorsed by the publisher.
