# Supplementary material for: Differential Impact of COVID-19 Risk Factors on Ethnicities in the United States
Source: Front Public Health. 2021 Dec 6;9:743003. doi: 10.3389/fpubh.2021.743003 (PMC8687082; doi:10.3389/fpubh.2021.743003)
Supplement: Supplementary file 1 [file Data_Sheet_1.PDF]

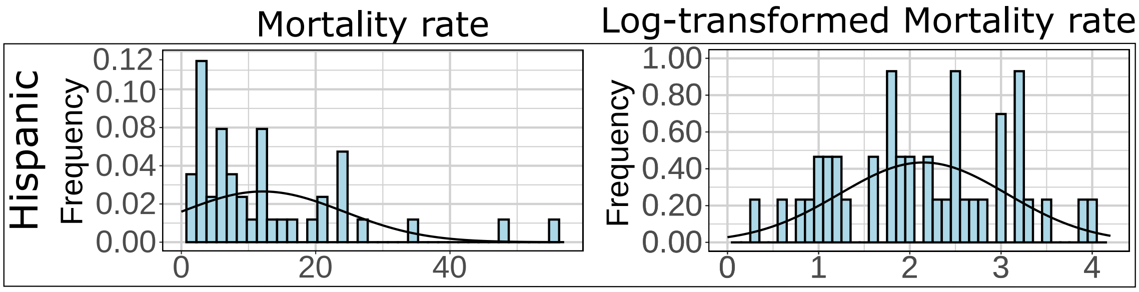

Figure S1. Normality plot for the mortality rate in the Hispanic group before and after log transformed response variable.

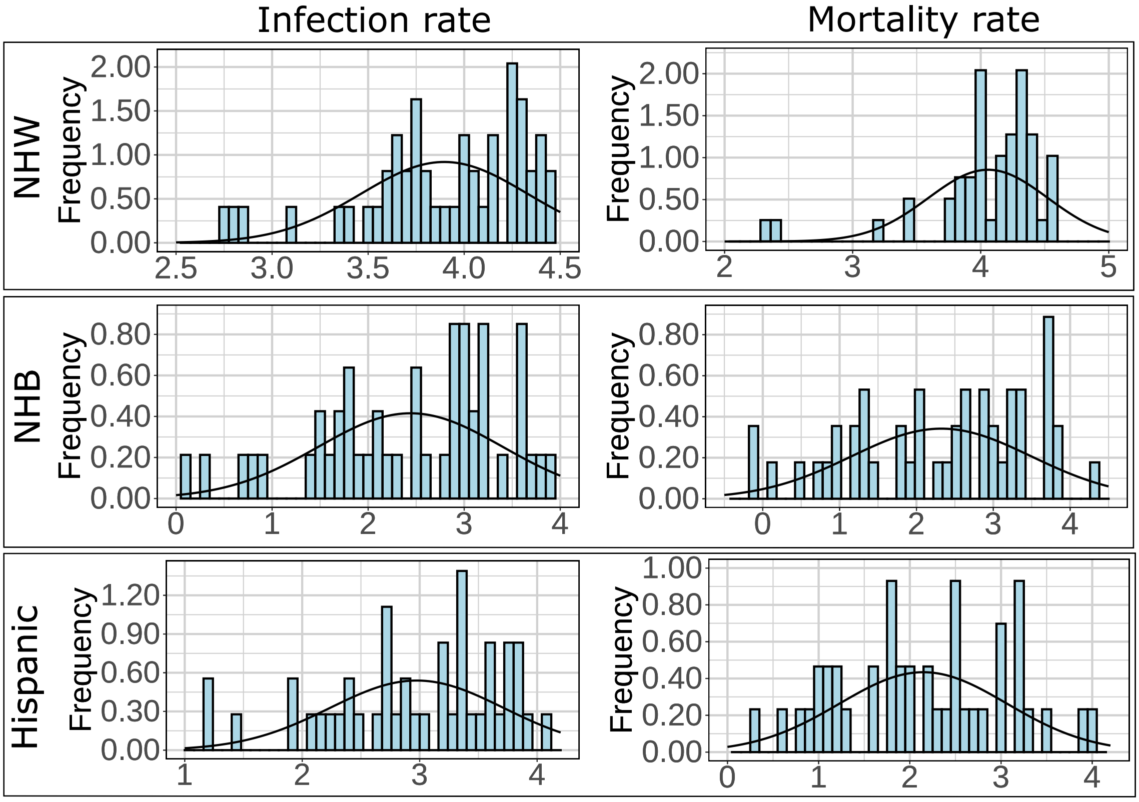

Figure S2. Normality plot of log-transformed infection and mortality rate for NHW, NHB, and Hispanics groups.

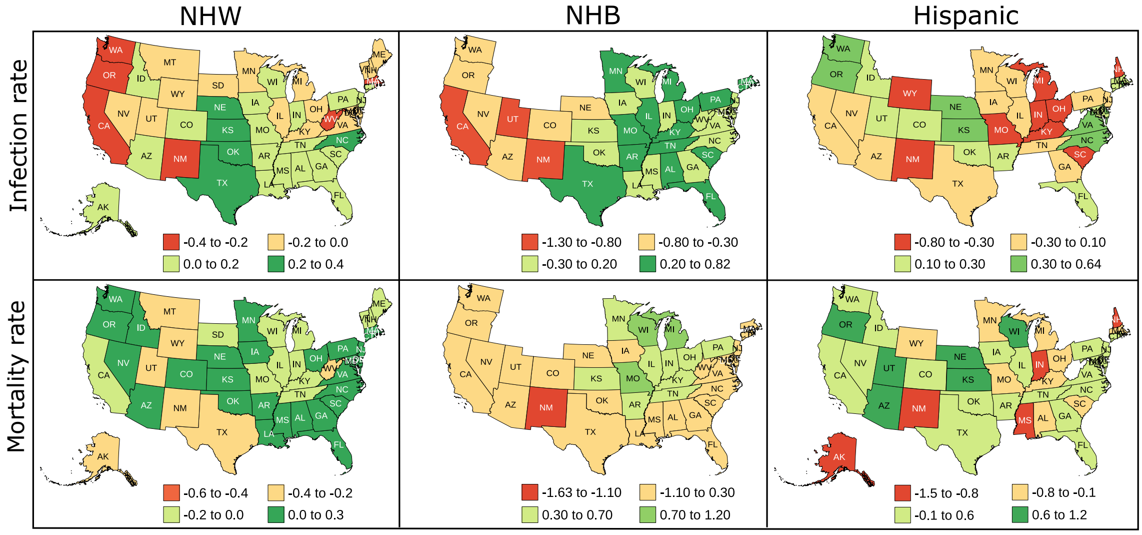

Figure S3. Residuals map for the log-linear regression for each ethnicity.

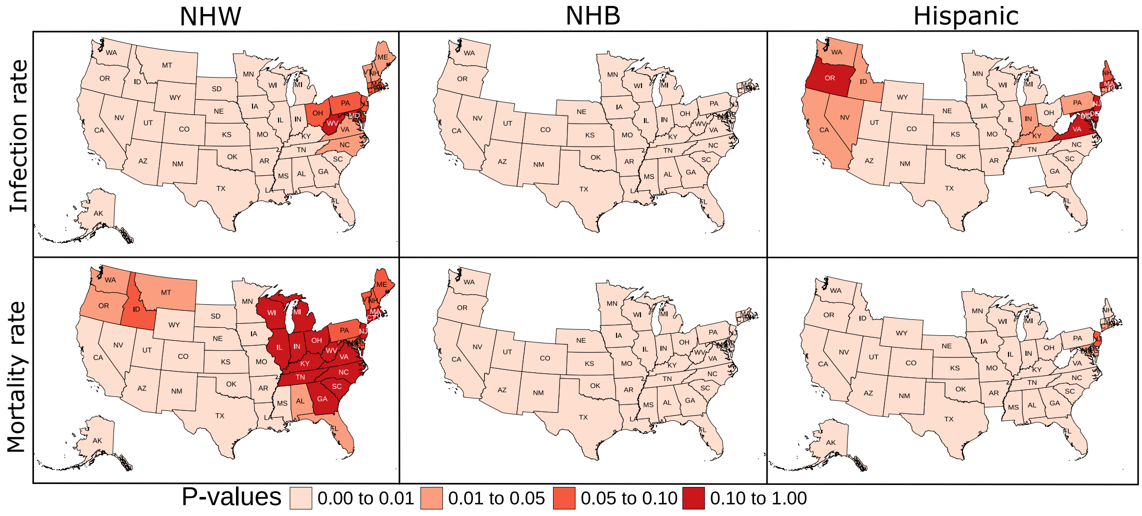

Figure S4. Maps of the p-values for the most significant coefficients obtained from geographically weighted regression,

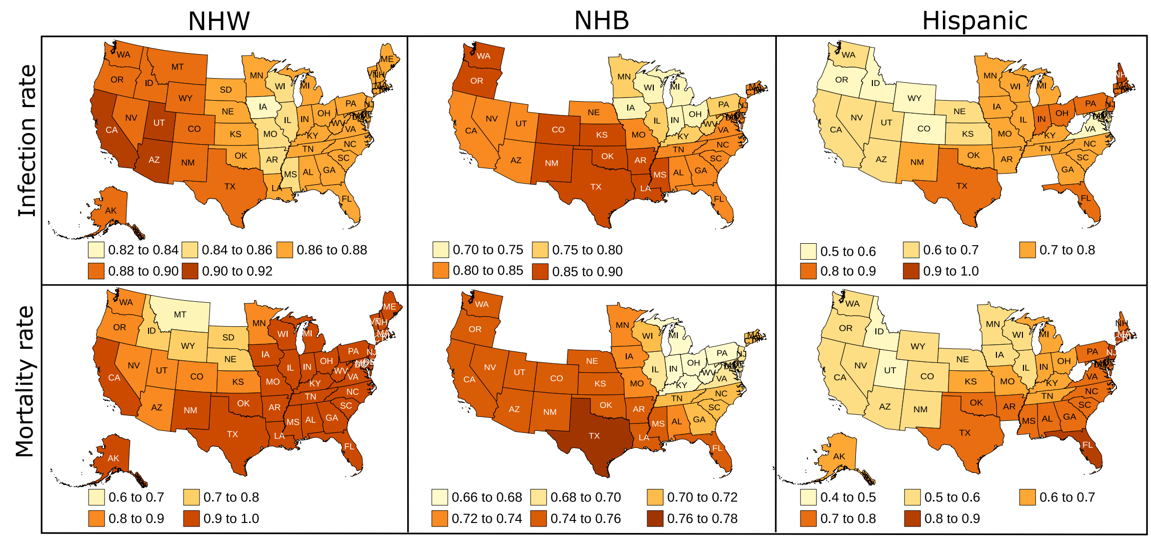

Figure S5. Local R^2 map for the geographically weighted regression.
